# Cognitive Strategy Use and Measured Numeric Ability in Immediate- and Long-Term Recall of Everyday Numeric Information

**DOI:** 10.1371/journal.pone.0057999

**Published:** 2013-03-06

**Authors:** Douglas Bermingham, Robert D. Hill, Dan Woltz, Michael K. Gardner

**Affiliations:** Department of Educational Psychology, University of Utah, Salt Lake City, Utah, United States of America; Western University of Health Sciences, United States of America

## Abstract

The goals of this study were to assess the primary effects of the use of cognitive strategy and a combined measure of numeric ability on recall of every-day numeric information (i.e. prices). Additionally, numeric ability was assessed as a moderator in the relationship between strategy use and memory for prices. One hundred participants memorized twelve prices that varied from 1 to 6 digits; they recalled these immediately and after 7 days. The use of strategies, assessed through self-report, was associated with better overall recall, but not forgetting. Numeric ability was not associated with either better overall recall or forgetting. A small moderating interaction was found, in which higher levels of numeric ability enhanced the beneficial effects of strategy use on overall recall. Exploratory analyses found two further small moderating interactions: simple strategy use enhanced overall recall at higher levels of numeric ability, compared to complex strategy use; and complex strategy use was associated with lower levels of forgetting, but only at higher levels of numeric ability, compared to the simple strategy use. These results provide support for an objective measure of numeric ability, as well as adding to the literature on memory and the benefits of cognitive strategy use.

## Introduction

Encoding and retrieving numbers from memory is an important capability in a technologically driven society. Examples of to-be-remembered number strings include computer passwords and unique personal identifiers such as personal identification and social security numbers. Memorizing and recalling information exactly, such as security numbers, can tax cognitive resources and requires substantial effort [1], [2]. Several factors including an individual’s facility and expertise with numbers enhance how numerical information is learned and subsequently retrieved [3], [4]. Research has consistently found that using cognitive strategies improves immediate and long-term recall [5], [6]. Therefore, exploring how individuals engage in memorizing every-day number information will assist with understanding, at a fundamental level, how strategies and numeric abilities enhance number-learning outcomes as separate and interactive processes.

### Cognitive Strategy Use and Mnemonics

Cognitive strategies, sometimes known as mnemonics, are techniques that enable individuals to memorize and recall new information in an easier fashion than would normally be the case in using rote rehearsal [7]. Training individuals to use cognitive strategies facilitates learning of new information across a wide range of ages and content domains. Several reasons have been proposed for the effectiveness of cognitive strategies over rote rehearsal. One involves the allocation and use of attentional resources to optimize cognitive resources [8]–[10]. That is, a strategy promotes more organized encoding that ultimately reduces the need for attentional resources at the point of retrieval [11]. A separate proposition made by Baltes [12] posits that leveraging semantic connections, such as those that link an unfamiliar number string with semantic cues (e.g. “In 1492 Columbus sailed the Ocean blue”), bootstraps episodic recall by activating cognitive reserve capacity.

The use of mnemonics has a well-documented history in the memory and learning literature including in early educational settings where organizational techniques (e.g. sorting) have shown demonstrable gains over rote rehearsal [13], [14]. This history encompasses the domain of numbers. For instance, the strategy of decomposition, or breaking large numbers down into smaller and easier to manipulate units or chunks [15] has been found to be effective for later recall of numbers and math facts. Through chunking one can also link semantic cues like familiar historical dates or times to the numeric chunk (e.g. 1492) [16]. Semantic memory is activated, thus facilitating long-term retention.

Training individuals in the use of formal cognitive strategies, such as the number-consonant mnemonic or the method of loci, has been shown to diminish even age-related differences in episodic recall [17]-[21]. This research parallels the more extended findings that other researchers have produced when older adults were trained to expert proficiency in the method of loci for the encoding and retrieval of verbal stimuli and age-related deficits were fully mediated through this process [22], [23].

Even in the absence of formal training, learner-generated strategies have been advantageous for later recall when compared to rote rehearsal. With regard to number strings, Hill, Schwob, and Ottman [20] found that self-generated strategies for recall of two-digit prices and four-digit phone numbers was associated with better outcomes at immediate and delayed recall intervals. These findings were later replicated by Derwinger, Stigsdotter-Neely, and Bäckman [18].

### Expertise

Expertise occurs when extended training and practice within a particular domain results in enhanced learning, including encoding and retrieval, of information [7]. Individuals who practice to become experts in a specific content domain learn to make more and deeper connections between to-be-learned information, which then assists them with encoding and retrieval [24].

Experts commonly employ cognitive strategies to assist in learning new information. Among chess masters, memory for chess positions is one example of domain-specific expertise [25], [26]. Strategic chunking of sports information is frequently used by sports professionals to encode and retrieve details such as baseball statistics [27], and hockey facts [28]. In addition, mental representations of music have been examined in expert versus novice musicians [29], as has pilot communication and memory for flight routes [30]. A hallmark of experts is the tailored use of cognitive strategies to facilitate the encoding and recall of information within the domain of expertise.

Experts are also non-professionals who have developed specific encoding and recall routines over a lifetime of practice. Castel [3] found that older shoppers could remember grocery prices just as well as less experienced shoppers due to the fact that a lifetime of shopping allowed older shoppers to rely on schema of commonly occurring items and prices that facilitated more accurate recall. In separate but related research, Castel [4] examined performance differences in older number experts (defined as accountants and bookkeepers) versus younger and older non-experts and found that, during a cued recall task, older experts outperformed the younger and older nonexperts. Castel suggested that because experts process information by accessing domain-relevant schema or use strategies unavailable to the nonexperts, that this skill mediates age-related recall deficits.

### The Present Study

A wealth of research indicates that expertise or extensive knowledge within a domain can enhance the recall of information within that domain [3], [4], [25]–[30]; separate research indicates that individuals who use cognitive strategies are able to recall information with more accuracy than those individuals who do not use strategies [7], [13]–[24]. What has not been examined in detail is if these two factors have an interactive moderating relationship. A moderation interaction indicates that the relationships between two variables (e.g. the use of cognitive strategies and recall of numeric information) can vary based on the level of a third variable (e.g. numeric ability). It may be that the beneficial effect of strategy use on recall of numeric information is moderated by an individual’s facility with numeric information. Furthermore, it is likely that either strategy use or numeric ability can have enhancing effects above and beyond immediate recall intervals; that is, these variables, separately or combined, may enhance overall recall, as well as the amount of retention over longer-term intervals.

**Figure 1:**
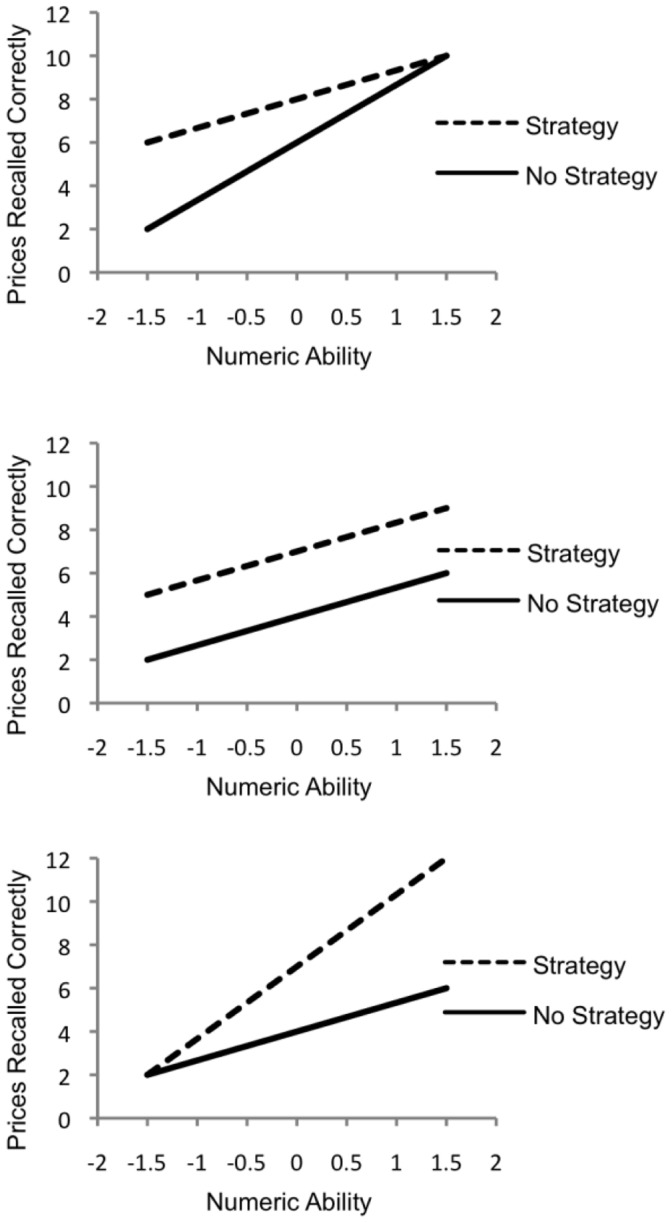
Possible Effects of Numeric Ability and Strategy Use on Recall of Prices. Three possible models of the effects of numeric ability and strategy use on recall of prices. Top: under-additive model, wherein the positive impacts of strategy use are reduced at higher levels of numeric ability. Middle: additive model, wherein strategy users and non-strategy users benefit equally from higher levels of numeric ability. Bottom: over-additive model, wherein the benefits of strategy use are enhanced at higher levels of numeric ability.

This study examined the role of measured numeric expertise (numeric ability) and the use of cognitive strategies as predictors of everyday number recall (e.g. prices) over a 7-day period. We hypothesized that: 1) scores on a composite measure of numeric ability would enhance short and long-term recall of numeric information; and 2) that the use of cognitive strategies would enhance short and long-term recall of numeric information. In addition, we wanted to examine the moderation that numeric ability would have on the relationship between strategy use and memory for prices. Therefore, three possible moderating interactions were investigated, based on the variables in this study. The first model predicts an under-additive numeric ability × strategy use interaction, in which the beneficial effects of strategy use on recall of information are reduced at high levels of numeric ability, relative to lower levels of numeric ability. The second model predicts additive effects of numeric ability and strategy use on recall; that is, there would be differences in recall between those who do and do-not use cognitive strategies, but both of these groups would benefit equally as numeric ability increases. Finally, the third model predicts an over-additive numeric ability × strategy use interaction, in which the beneficial effects of strategy use on recall of information are enhanced at high levels of numeric ability, relative to lower levels of numeric ability. Figure 1 outlines these three possible interactions graphically.

Overall, we wanted to evaluate the simple and joint effects of numeric ability and the use of cognitive strategies on memory for every-day numeric information (prices). Specifically, we wondered if the effects of numeric ability and cognitive ability on memory for prices were additive or interactive over a 7-day interval.

## Methods

### Ethics Statement

This study adhered as strictly as possible to University of Utah ethical policies, as well as local, state and national laws relating to human subjects research. The study protocols, design, and recruitment techniques were approved by the University of Utah’s Institutional Review Board (approval #IRB_00030432). Written consent was obtained from all participants immediately prior to their beginning the study.

### Participants

Participants were 112 individuals recruited from the University of Utah Educational Psychology Subject Pool, The University of Utah Alumni Online Newsletter, and the University of Utah OSHER Life Long Learning Institute. Individuals were a mixture of full-time students, employed professionals, and retired persons. Many students participated in this research for course credit in an introductory Educational Psychology course. Community-dwelling participants were volunteers. Among these persons, 77 were female, 32 were male, and three chose not to disclose their gender. Ages ranged from 18 to 69, with a mean age of 27 years (*SD* = 10.6 years). All reported completing some college education (*M* = 13 years education, *SD* = 5.9 years).

During the initial data screening the distribution of ages was negatively skewed with only five individuals over the age of 53, ranging up to 69 years; therefore, these five cases were dropped to provide more homogenous age data. Similarly, seven severe outliers on the dependent variables and numeric ability variable were identified with Mahalanobis distances that were significant (χ^2^(3) = 16.3, *p*<.001) and were therefore dropped, leaving a final sample of 100 participants. Among these individuals, 69 were female, 28 were male, and three chose not to disclose their gender. Ages ranged from 18 to 53, with a mean age of 25 years (*SD* = 6.9 years). All reported completing some college education (*M* = 16 years education, *SD* = 1.58). These data are presented in Table 1.

**Table 1:** Descriptive Statistics for Measures of Numeric Ability, and Recall at Immediate and 7-day Post-test.

Variable	*M*	*SD*	Range
Age^a^	25	6.9	18–53
Years of Education^b^	16	1.58	12–
Addition^b^	14.99	5.12	0–30
Subtraction/Multiplication^b^	11.57	4.74	1–24
Division^b^	9.33	5.34	0–28
Correct/Incorrect^b^	45.43	14.7	1–97
Number Series^c^	11.55	5.11	0–19
Recall Time 1^d^	6.59	2.83	1–12
Recall Time 2^d^	2.55	1.74	0–6

a Sex coded as: 1 =  male, 2 =  female.

b Based on a total of 60 items.

c Based on a total of 20 items.

d Based on a total of 12 correct prices.

### Measures

Numeric ability was assessed using the Number Facility subtests (number facility forms 1–4) from the Kit of Factor-Referenced Cognitive Tests (French Kit) [31] and the number-series subtest of the Cognitive Abilities Test (CogAT) [32]. Given that these subtests were all significantly related, the objective was to combine them into a unitary index of numeric ability; if they were not related, separate subtests would be used to assess numeric ability.

The French Kit contains 72 tests that measure a broad array of cognitive processes including verbal ability, reasoning, spatial ability and memory, and is often used in cognitive research [31], [33]–[34]. The four French Kit number facility tests require participants to supply answers to questions about mathematical operations, and include subtests assessing addition, subtraction/multiplication, division, and the ability to recognize correct or incorrect answers given for a particular mathematical operation. The addition subtest is a speeded test that requires participants to provide answers to sets of three 1- or 2-digit addition problems. The subtraction/multiplication subtest that alternates between 10 items requiring the subtraction of 2-digit numbers from 2-digit numbers, and 10 items requiring the multiplication of 2-digit numbers by 1 digit numbers. The division subtest involves dividing 2 or 3 digit numbers. The correct/incorrect problems ask the participant whether an answer shown for a particular subtraction or addition problem is correct or incorrect. All subtests are 60 items long, divided into two pages of 30 items, and each page is timed for 2 minutes. Items are scored correct if the correct response is given; no points are given for incorrect answers. Subtest scores are sums of all items for that subtest, and are not normed; an individual’s raw score is used as an indicator of number facility. Scores from the French Kit can be used to derive an index of an individual’s ability to perform mathematics accurately and quickly, which estimates number facility (cognitive factor *N*). The number facility tests have been reported to be internally consistent with Cronbach alpha coefficients ranging from.86 to.94 as reported in the test manual [31].

The number series subtest from the CogAT was chosen as a compliment to the French Kit mathematics tests as it requires the manipulation of numeric information and is a good measure of quantitative fluid reasoning abilities [32]. Research suggests that different cognitive processes are involved in the recollection of basic math facts versus the manipulation of numeric information involved in more fluid quantitative reasoning [35]. The CogAT has several different forms based on the grade level of the individual being tested; Form H was the form used during this study, as it was the closest in relation to the age of the individuals being tested. The number-series subtest presents the participant with a series of numbers, asks the participant to determine the “rule” governing the progression of the series, and to provide the next number in the series. For instance, one question provides the participant with the following sequence of numbers: 2, 6, 3, 9, 4; the participant is then asked to select an answer from among four options (the correct answer being 12). There are twenty items total, increasing in complexity, and the test is timed for 10 minutes. Items are scored correct if the correct response is given; no points are given for incorrect answers. Although the CogAT does provide normative data for children (K-12 education), normed scores were not utilized; rather, raw scores were used as measures of quantitative reasoning ability. No internal consistency estimates were reported for the number series subtest in the technical manual; however, the quantitative battery (of which the number series is a subtest) has a reported alpha coefficient of.94. The CogAT has been traditionally used to assess reasoning abilities of individuals in elementary and post-elementary educational settings (grades K-12), yet there is convergent validity evidence between the Multilevel Battery of the CogAT (Quantitative, Verbal and Nonverbal reasoning) and a combined score from the Woodcock Johnson-III General Ability composite, which has often used to assess adults outside of an educational setting (*r* = .69).

The memorization and recall task involved the presentation of a series of 12 prices that were created using a random number algorithm. The numeric stimuli of prices were chosen for several reasons: first, prices are commonly occurring numbers with real life applications and relevance. Second, they are readily associated with some stem or cue. Finally, Castel [26] as well as Hill, Schwob and Ottman [15] used prices as a stimulus material for number-recall. These prices ranged from one digit ($7) to six-digits ($447,289), and were always rounded to the nearest dollar amount. This range in digit length was chosen to prevent floor and ceiling effects from occurring; in Castel’s study [28], some floor effects were observed using 4-digit numbers. Items that might realistically be purchased for each price were found using an internet search, and paired with the appropriate price (e.g. a Yacht could match the generated price of $769,140), thus forming a realistic pair of cues and stems. Realistic associations between each cue-stem pair were developed to prevent atypical and distinctive pairings (e.g. Pickup: $5) that could potentially be easier to memorize due to their uniqueness. This methodology used to create the cue-stem pairs was chosen for two reasons: First, market prices for actual items can fluctuate based on a number of factors that were out of the control of this study (e.g. price of gasoline can inflate costs due to shipping). Second, the current study was more interested in assessing how numeric ability impacted recall, rather than a schema of actual/market prices developed over one’s lifetime. In this way, this study diverged from Castel’s [27] research on schematic support as a form of domain knowledge and recall of prices. All cue-stem pairs are presented in Table 2.

**Table 2:** Cue-Stem Pairs, and Frequency Recalled at Immediate and 7-day Post-Tests.

Cue	Stem	Frequency Time 1	Frequency Time 2
Paperweight	$5	96	68
Scissors	$7	95	67
Purse	$36	79	41
Backpack	$87	84	32
Painting	$395	63	13
Stereo	$645	57	11
Hot Tub	$1,472	51	8
Computer	$2,364	36	6
Car	$38,986	26	4
Pickup	$52,864	26	2
House	$477,289	26	2
Yacht	$769,140	20	1

During the recall task, the cues were presented in a random order, and participants were queried for the correct stem. The presentation of the cues in a random order was to prevent the participant from having memorized the order of the pairs, rather than the cue-stem pair itself. Each item was scored correct if the participant provided a verbatim answer; no points were given for stems that were close to correct. The amount of time the participant took during the recall task was not recorded. Demographic information was also collected from participants including age, sex, and years of education attained.

Strategy use was assessed using an open-ended question and form. Participants were asked to “Please list and describe any methods you used to remember the prices you memorized earlier.” This self-report was not timed.

### Procedures

All measures were presented via a computer interface located in a laboratory setting that was quiet and provided participant privacy during the learning and recall phases. At the immediate recall interval, the participant was seated in front of the computer and presented with a description of the study along with both a digital and a paper version of the participant consent form. The participant was then presented with a screen that introduced the first memory task, which involved a practice task. A practice list of 7 cue-stem pairs (not used in the actual experiment to follow) were displayed on the screen which the participant was given 4 minutes to study, after which time a practice recall page was presented. The participant was given an unlimited amount of time to recall the practice cue-stem pairs before submitting the answers. During the actual memory task that followed, each participant was presented with the complete list of 12 cue-stem pairs. Seven minutes was allotted to study this list, and participants appeared to use the entire time to study; no participants were observed to engage in other tasks during this time. Study and recall time intervals were established based on those reported by Hill, Schwob and Ottman [15].

Following the seven minute time allotment, the participant completed an on-line demographic questionnaire. Directly following the demographic questions, the participant read several paragraphs on unrelated topics (e.g., a story about baboons) until a 10-minute time interval elapsed. The use of the demographic questionnaire and 10-minute time limit was to prevent the participant from rehearsing the cue-stem pairs, thus acting as a distracter task. The participant was then presented with the recall task. This consisted of the presentation of the entire list of cues arranged in a random order. The recall task involved entering numbers to replace blanks with the correct stem that corresponded to the cue (e.g., Stereo: $ __ __ __ __ __ __”). Participants were given an unlimited amount of time during the recall task. At the conclusion of the recall task, the participant was asked to complete the mathematical proficiency section of the French Kit, followed by the number series test from the CogAT. Follow-up instructions were provided about how and when to return for re-testing after 7-days had passed. Participants were instructed not to write the stems down or to spend time rehearsing the cue-stem pairs during the 7-day interval. Upon returning for 7-day re-testing, the participant was seated and the recall task was administered using the same processes outlined for the immediate post-test. At this time they were also asked to detail, using an open-ended form, any cognitive strategies they used to assist with recalling the cue-stem pairs. Asking for this self-report at the 7-day post-test, rather than at the immediate post-test, was to prevent priming the participant that he or she should use a cognitive strategy. In this way, hopefully the participant would give an accurate assessment of the methods they used to encode and recall the cue-stem pairs.

## Results

Descriptive statistics for the French Kit Number Facility subtests, the CogAT number series subtest, and recall of the cue-stem pairs at immediate (Time 1) and 7-day delayed recall (Time 2) are presented in Table 1. As can be seen, the participants represented a wide range of ability on the number facility and number series tests, as well as ranging in their ability to recall the cue-stem pairs at Time 1 and Time 2. As would be expected, recall was higher at Time 1 when compared to Time 2. Thirteen individuals scored at the floor (0 stems recalled) at Time 2; no floor effects were observed at Time 1. Five individuals recalled all 12 stems correctly at Time 1, while no one was able to recall all 12 stems at Time 2. The smaller stems (e.g. 1–2 digits) were easier to recall compared to the larger stems (e.g. 5–6 digits) at both Time 1 and Time 2. Distributions of stems were both positively skewed (smaller stems being easier to recall), with the skew at Time 2 being more prominent (see Table 2).

Internal consistency estimates were calculated for the French Kit Number Facility subtests as well as the CogAT number series subtest, and recall at both immediate (Time 1) and delayed (Time 2) follow-up. French Kit subtest internal consistency coefficients were adequate to good (Cronbach alphas: Addition = .77, Subtraction/Multiplication = .75, Division = .81, Correct/Incorrect = .85), while the CogAT showed good internal consistency with a Cronbach alpha of.89. Recall at Time 1 had good internal consistency (Cronbach alpha = .80), while Recall at Time 2 had lower internal consistency (Cronbach alpha = .61).

Answers from the open-ended strategy use questionnaire were analyzed by three independent raters and categorized based on complexity of the strategy use described on a scale of 0–4, with 0 being no strategy used, and 4 being a highly complex strategy, such as a formalized mnemonic (e.g. number-consonant) or the integration of several complicated strategies. Ninety-six people provided answers to this question, with four abstaining. Individuals who responded as having used no strategy (e.g. “I tried my hardest”) were coded as No Strategy. Those who used the strategy of repetition (e.g. “I repeated the numbers to myself over and over until the time ran out”) were coded as Repetition. Those who used a single cognitive strategy (e.g. self-referencing: “I thought about how much I have paid for some of the items, when I have bought them myself. I also thought about if the price was a price I would pay;” self-testing: “I tested myself with each item. I would try not to look at the price but just the name of the item and recall the price by memory and then check to see if it was right. I did this continuously until the time was up”) were coded as Elaborative Strategy. Finally, those who used several cognitive strategies, or who used a formalized mnemonic (e.g. “For a few of the prices, I remembered how the prices looked on the 10-key pad (1472 and 2364). Scissors was easy to remember because 7 starts with “s” just like scissors. All the lower priced items were easy to remember - 3 is half of 6 (for 36). 87 is in decreasing order”) were coded Complex Strategy. Agreement on the ratings was reached by consensus among the raters. During initial data screening the No Strategy category was noted to have a very small frequency (n = 5) and that the distribution of ratings between the other categories were quite uneven (Repetition = 17, Simple Strategy = 64, Complex Strategy = 10). Because of these discrepancies, and to facilitate analysis, the strategy use variable was collapsed into a dichotomous variable: Strategy v.s. No Strategy used. Repetition was considered to be non-strategic in comparison to the cognitive strategies reported. Of the 94 respondents, 22 used no strategy and 74 used a strategy.

For all statistical tests an alpha level of.05 was used. Pearson correlations were computed for age, sex, education, the French Kit Number Facility subtests, the CogAT Number Series test, and recall at Time 1 and Time 2 (See Table 3). Age and years of education were not significantly related to recall at Time 1 or Time 2. Age was not related to any of the numeric ability tests; however, education was negatively related to the subtraction/multiplication subtest, the correct/incorrect subtest, and the number series subtest. The use of strategies was positively related to the addition subtest and the number series subtest, but no other measures of numeric ability. The use of strategies was also weakly related to recall at both Time 1 and Time 2.

**Table 3:** Pearson correlation matrix.

Variable	1.	2.	3.	4.	5.	6.	7.	8.	9.	10.	11.
1. Age											
2. Sex^a^	.08										
3. Years Education	.26**	.03									
4. Addition	−,07	−.15	−.12								
5. Subtraction/Mult.	.10	−.21*	.03	.57**							
6. Division	.06	−.11	−.05	.35**	.67**						
7. Correct/Incorrect	−.05	−.35**	−.04	.49**	.73**	.55**					
8. Number Series	−.18	−.31**	−.10	.33**	.46**	.44**	.69**				
9. Numeric Ability	−.03	−.29**	−.06	.68**	.88**	.77**	.89**	.73**			
10. Strategy Use^b^	−.19	−.15	.05	.25**	.06	.11	.12	.31**	.21*		
11. Recall at Time 1^c^	−.09	−.04	−.01	.13	.27**	.21*	.21*	.30**	.32**	.25*	
12. Recall at Time 2^c^	−.08	.08	.02	.25*	.33**	.30*	.30*	.29**	.39**	.21*	.51**

* *p*<.05, ** *p*<.001.

a Sex coded as: 1 =  male, 2 =  female.

b Strategy use coded as: −1 =  no strategy used, 1 =  strategy used.

c Based on a total of 12 correct prices.

Subtest scores from the French Kit and the CogAT were all significantly related, ranging from moderate to strong relationships. A first order principle components extraction was employed to verify the assumption that these tests were measuring a similar construct, and all five subtests loaded on a single factor suggesting that using a composite to measure a unitary factor was appropriate. Therefore, to create this numeric ability index, measured scores from the French Kit and the CogAT were transformed into a composite through the first order principle components extraction, and this component score was used in future analyses.

To address the primary hypotheses and test the proposed moderation models, the effects of recall occasion, numeric ability, and strategy use were tested with a repeated measures analysis of covariance. The two testing occasions represented the repeated measure dependent variable. These two measures were transformed with orthogonal contrasts to represent the average recall (i.e., an overall recall score) and the difference between Time 1 and 2 (i.e., forgetting). Self-reported strategy, coded −1 (no strategy) and 1 (strategy), was a between group factor. Finally, the numeric ability variable and its interaction with strategy use were covariates. Main effects for numeric ability and strategy use were predicted for both the average recall and forgetting contrasts. In addition, the Strategy × Numeric Ability interaction tested the prediction that numeric ability moderated the impact of strategy use on memory for prices.

**Figure 2:**
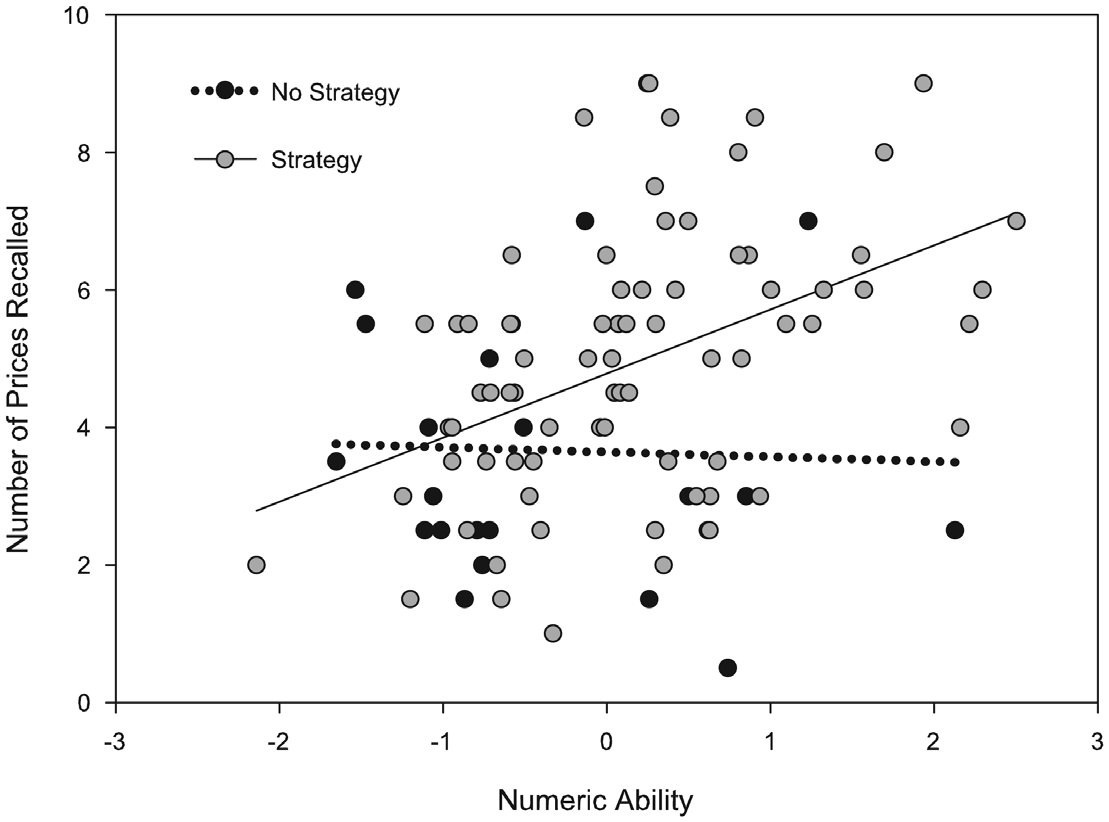
Moderating Interaction of Strategy Use on Numeric Ability for Overall Recall. Overall recall regressed onto numeric ability for the two strategy groups (no strategy used v.s. strategy used). A significant moderating interaction of strategy use was found on the relationship between numeric ability and overall recall of prices (recall averaged over the immediate and 7-day recall intervals). Self-reported strategy use was associated with better overall recall only at higher levels of numeric ability. Numeric ability is represented as a mean-centered variable.

As expected, there was a large main effect of test time, *F*(1, 92) = 145.96, *p*<.001, *η_p_* ^2^ = .61. As seen in Table 1, recall performance declined substantially over the 7-day delay. The effect of numeric ability on average recall approached but did not reach statistical significance, *F*(1, 92) = 3.62, *p* = .06, and its effect on the forgetting contrast was also non-significant, *F*(1, 92) = .34, p>.05. Self-reported strategy use had a small but significant relationship with average recall, *F*(1, 92) = 6.29, *p* = .01, *η_p_* ^2^ = .06, and no relationship with forgetting, *F*(1, 92) = 1.62, *p* = .21. As seen in Table 4, those reporting strategy use recalled slightly more prices overall than those who did not. Finally, and of primary importance, there was a statistically significant interaction between strategy use and numeric ability for explaining average recall, *F*(1, 92) = 4.89, *p* = .03, *η_p_* ^2^ = .05, but not for explaining the forgetting contrast, *F*(1, 92) = .27, p>.05. Figure 2 contains a scatter plot of average recall regressed on numeric ability for the two strategy groups. The different slopes correspond to the significant Strategy × Numeric Ability interaction. As seen in this figure, self-reported strategy use was associated with better overall recall only at higher levels of numeric ability.

**Table 4:** Overall Recall and Forgetting of Prices by Strategy Use.

Recall and Strategy Type			*M*				*SD*		Range				
Overall Recall^a^													
Strategy Used			4.93			1.88					1–9		
No Strategy Used			3.66			2.10					.5–9		
Simple Strategy Used		4.62			1.71				1–9				
Complex Strategy Used	6.95			1.77				1–9					
Forgettingb													
Simple Strategy Used	3.89			2.37				−1–9					
Complex Strategy Used	5.90			2.13				2–9					

a Overall recall was calculated as an average of prices recalled from immediate post-test to 7-day post-test.

b Forgetting was calculated as the difference between prices recalled from immediate post-test to 7-day post-test. A negative value would indicate more items were recalled at the 7-day post-test than were recalled at the immediate post-test.

As a supplementary exploratory analysis those individuals who reported using more complex strategies (e.g. mnemonics) were compared to those who used simpler strategies (e.g. self-testing) in a second repeated measures analysis. To accomplish this, those individuals who reported using no strategy (n = 22) were excluded from the analysis. The original coding schema (simple strategy v.s. complex strategy) was reinstated, resulting in 64 individuals using simple strategies, and 10 individuals using complex strategies. The effects of recall occasion, numeric ability, and strategy use were tested with a second repeated measures analysis of covariance. The two testing occasions represented the repeated measure dependent variable. These two measures were transformed with orthogonal contrasts to represent the average recall (i.e., an overall recall score) and the difference between Time 1 and 2 (i.e., forgetting). Self-reported strategy, coded −1 (simple strategy) and 1 (complex strategy), was a between group factor. Finally, the numeric ability variable and its interaction with strategy use were covariates.

**Figure 3:**
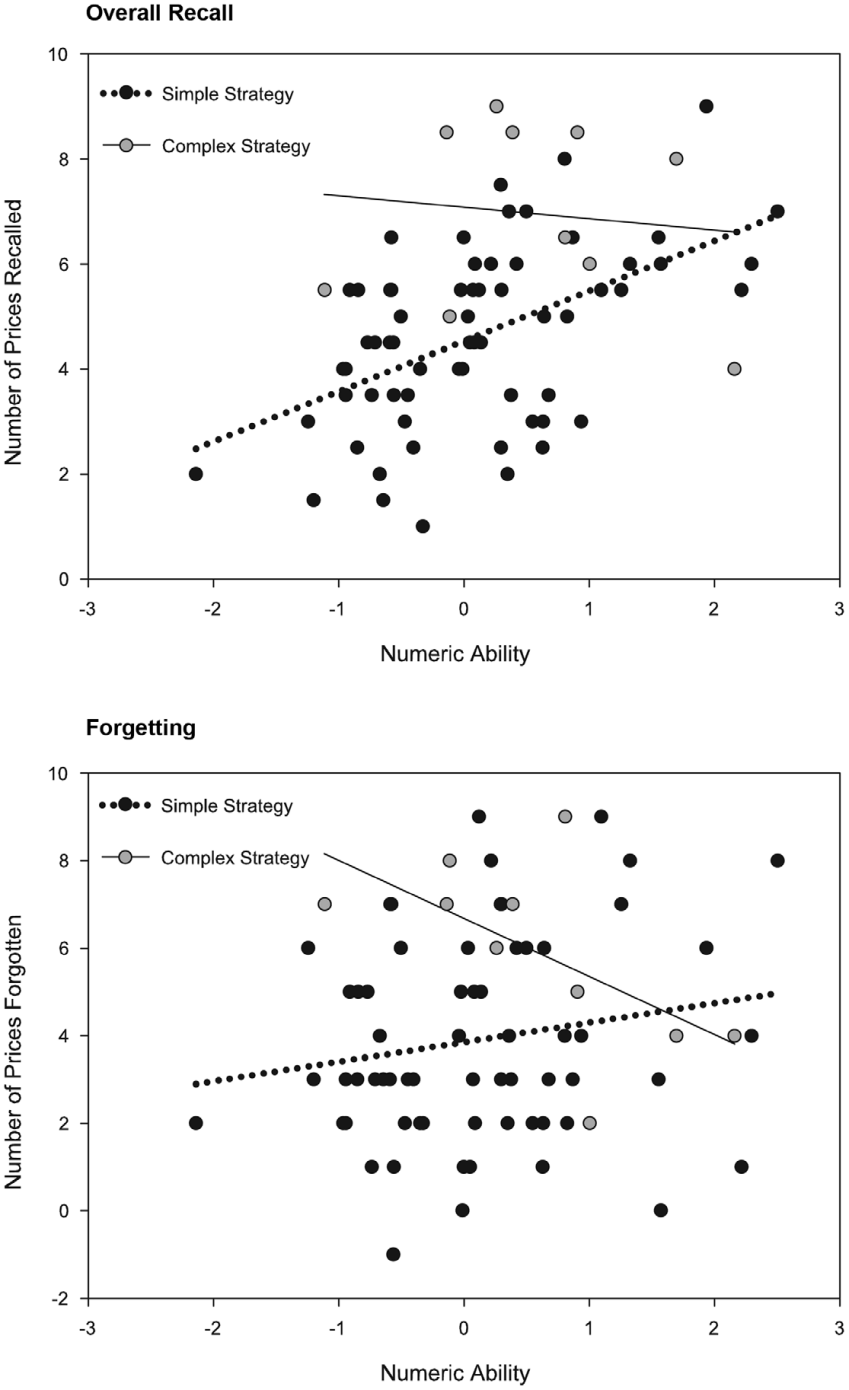
Moderating Interaction of Strategy Use on Numeric Ability for Overall Recall and of Forgetting. Overall recall and forgetting regressed onto numeric ability for the two strategy groups (simple strategy v.s. complex strategy). A significant moderating interaction of strategy type was found on the relationship between numeric ability and overall recall of prices (recall averaged over the immediate and 7-day recall intervals). Simpler strategy use was associated with better overall recall only at higher levels of numeric ability for average recall. A significant moderating interaction of strategy type was also found: more complex strategies were associated with lower rates of forgetting, but only at higher levels of numeric ability. Numeric ability is represented as a mean-centered variable.

Self-reported strategy use had a significant relationship with average recall, *F*(1, 70) = 17.66, *p*<.01, *η_p_* ^2^ = .20, and a significant relationship with forgetting, *F*(1, 70) = 9.57, *p*<.01, *η_p_* ^2^ = .12. As seen in Table 4, those reporting complex strategy use recalled slightly more prices overall than those who used simpler strategies, but tended to forget prices more readily. Finally, and of primary importance, there was a small but statistically significant interaction between strategy use and numeric ability for explaining average recall, *F*(1, 70) = 4.13, *p* = .046, *η_p_* ^2^ = .06, as well as for the forgetting contrast, *F*(1, 70) = 4.17, *p = *.045, *η_p_* ^2^ = .06. Figure 3 contains scatter plots of average recall and forgetting regressed on numeric ability for the two strategy groups. The different slopes correspond to the significant Strategy × Numeric Ability interaction. As seen in this figure, simpler strategy use was associated with better overall recall only at higher levels of numeric ability for average recall; and more complex strategies were associated with lower rates of forgetting, but only at higher levels of numeric ability.

## Discussion

The goals of this study were to assess the primary effects of the use of cognitive strategy, and a combined measure of numeric ability on recall of every-day numeric information (i.e. prices). Additionally, we examined whether numeric ability would moderate the relationship between strategy use and memory for prices. In doing so, we tested three possible moderating models: an under-additive model in which strategy use would be more effective at lower levels of numeric ability; an additive model in which numeric ability would enhance recall for both strategy users and non-strategy users equally; and finally an over-additive model in which strategy use is most enhanced at high levels of numeric ability. We analyzed memory as an overall construct (e.g. memory averaged across immediate and 7-day post-tests) as well as rate of forgetting (e.g. difference between recall at immediate and 7-day post-tests).

We found strong significant relationships among the measures of numeric facility from the French Kit and the number series subtest from the CogAT, and therefore combined them into a unitary index of numeric ability. Numeric ability was not significantly related to overall recall, or rate of forgetting. This is interesting as most other studies that measure domain expertise or domain knowledge have found significant, albeit sometimes small, effects of domain knowledge [20]–[26]. It is possible that the effect of numeric ability on overall recall or rate of forgetting is quite small, and would require a larger sample in order to detect a significant effect. Indeed, a post-hock analysis indicated that our tests of numeric ability were under-powered, even though they approached significance.

The main effect of strategy use, dichotomized as Strategy use vs. No strategy used, was found to be significant with respect to overall memory, but not with forgetting. This suggests that, in this study, individuals who used strategies had better overall recall at immediate and the 7-day delayed post-test, but that their rate of forgetting was not impacted by the use of strategies. This finding is consistent with other research which has found that the amount of information one recalls after using a cognitive strategy is enhanced [15], [19]–[21]. However, it does provide a contrast with other studies that have found lower rates of forgetting for individuals who used cognitive strategies compared to those who have not. Several studies that have trained individuals in the use of formalized mnemonics, or primed individuals to develop their own mnemonic strategies, have found either a lower rate of forgetting, or even a greater degree of recall at later recall intervals [19]–[21]. One likely reason for this discrepancy is that individuals in our study were self-reporting the strategies they used, rather than being primed to use a strategy, or instructed in the use of a formalized strategy per se.

While the preceding results add to the literature on the effectiveness of cognitive strategies, a more noteworthy discovery was a moderating effect of numeric ability on the relationship between strategy use and overall recall. The evidence from this study supports an over-additive model; that is the beneficial effects of strategy use on the recall of prices was enhanced at higher levels of numeric ability, relative to lower levels of numeric ability. Those individuals who used no strategies recalled about the same number of prices regardless of their numeric ability. This finding suggests that, for those individuals who do use cognitive strategies for the retention and recall of numeric information, having some facility with numbers is advantageous. It may be that familiarity with patterns of numbers, or rules of mathematics, could facilitate recall by way of number-based schema developed over time [27], [28]. It may also simply be that one’s experience with numbers and mathematics reflects an underlying propensity to process numerical information of all sorts more readily, and that individuals with this propensity are likely to use strategies that associate crystallized episodic information (e.g. important dates or times) with the newer, to be recalled numeric information [8].

As an exploratory step to better understand the observed moderating interaction, a second moderation analysis was performed, this time comparing simple and complex strategy users. Although the complex strategy users represented a very small sample, the results from this analysis were enlightening. The complex strategy users recalled more prices overall compared to the simple strategy users; however, they also tended to forget more of the prices at the 7-day post-test. Many studies that involves training individuals in formalized mnemonics - complex strategies, to be sure - have found that long-term recall is typically enhanced [16], [19]–[21]. One would expect that more complex mnemonics would allow individuals to retain more information (that is, forget less information) over longer periods of time. It may be that, for the small sample of complex strategy users in the present study, the complex strategies used allowed the individuals to encode the to-be-recalled information superficially but with enough resolution to provide good immediate recall, and that over the 7-day period the memory trace eroded more quickly. A second possibility is that over the course of the 7-day recall period memory for the strategy itself (or the cues produced by the strategy) had faded. The training literature indicates that complex strategies need to be over-learned to be truly effective in enhancing long-term recall [16]–[20].

Another noteworthy finding from this exploratory step was a second set of moderating interactions. Small moderating effects of numeric ability on the relationship between type of strategy, and overall recall, as well as forgetting, were observed. With respect to overall recall, an under-additive model was observed; that is, complex strategy use was not enhanced by higher levels of numeric ability, relative to lower levels of numeric ability. To the contrary, simple strategy users were most benefitted by higher levels of numeric ability. This finding is rather surprising, as one might expect that higher levels of numeric ability would allow individuals to develop more complicated cognitive strategies, or enhance already known mnemonics, for better recall overall. Individuals with high levels of domain knowledge or expertise in other domains (e.g. chess masters or baseball statistics) have been known to tailor cognitive strategies based on their extensive bases of domain knowledge [21]–[26].

The second observed moderation indicated that complex strategy users tended to forget more prices when numeric ability was lower, but forgot less prices when numeric ability was higher, relative to the simple strategy users whose rate of forgetting remained relatively similar. These results suggest that the long-term impacts of complicated strategies are most enhanced when individuals are able bootstrap domain expertise or knowledge, while simple strategy users forget the same amount of information independent of numeric ability. Possibly, those individuals who used complex strategies and with greater levels of numeric ability, had surplus attentional resources to allocate to the memorization task thus enabling them to more deeply encode the prices [4], [6]. A second possibility is that those individuals with greater numeric ability were able to devote more of their cognitive resources to remembering the strategy used and the cues it provided. Assess to this information would be essential at posttest if the strategy were to enhance recall.

To be sure, these exploratory findings may have been artifacts of the small number of individuals reporting complex strategy use, as well as how strategy use was measured by self-report in the present study. Future research could extend these findings by training a larger sample of individuals to use complex strategies or formalized mnemonics, and compare their results to a group trained in the use of less complex strategies. Alternatively, future research could also further assess self-reported strategy use, as individuals who recalled more at the delayed interval may have been more motivated to explain their encoding in terms of a specific strategy and in greater detail than those who remembered less after the seven day interval. Obtaining accurate self-reports of strategy use has been highlighted in previous work [16]; therefore, we are currently engaged in developing a standardized approach for obtaining and evaluating strategy self-reports.

The present study improves on Castel’s [26], [27] methods by including a measure that quantified numeric ability. This measure operationalized numeric ability through a composite index score from two standardized number tasks; namely, knowledge of math facts measured by the French Kit number facility tests, and number-based reasoning ability measured by the CogAT number series subtest. Although the CogAT has not typically been used on individuals outside of an elementary or secondary educational setting, the results from the present study provide some support for using it on an older population. The French Kit number facility tests were significantly correlated with the CogAT number series subtest, but not so strongly as to suggest they were measuring the same construct. Additionally, the combined index of numeric ability that was created in the present study provides a broad-based measure of both crystallized knowledge of mathematics facts, as well as a fluid-based assessment of numeric reasoning.

Studies that have examined recall of numeric ability in older adults have found that, in some instances, older adults rely more on gist-based recall rather than attempting to recall the digits with exact precision as a form of aging-compensatory strategy [36]. Gist based-recall was not assessed in the present study because in many real-world situations precise digit recall is imperative. For instance, when recalling one’s social security number, a security PIN, or pass-code combination, being “close enough” is not sufficient. Similarly, accurately recalling one’s bank account balance prevents overdrafts when making withdrawals or charges in a shopping environment. Therefore, the present study provided an ecologically valid assessment of memory for every-day numeric information. The impact of aging was also not a central focus of the present study, primarily due to the attenuated range of ages of the participants. Future explorations into the interaction of numeric ability and strategy use could employ a larger sample of older participants, and in turn assess gist-based recall as a strategy use category.

One major variable that was not assessed in the present study was working memory capacity (WMC). Several noteworthy studies have found that domain knowledge can enhance recall for domain specific information, but that WMC has a markedly larger impact [37], especially when considering the negative effects of older-age [23]. This highlights a limitation in the present study in that WMC was not assessed, and presents an opportunity to examine to what degree WMC might moderate the relationships between numeric ability, strategy use, aging, and recall of numeric information.

In conclusion, the present study found that a composite numeric ability measure based on mathematical facility and number reasoning interacted with the use of cognitive strategies to enhance recall of numeric information. Exploratory analyses found that greater levels of numeric ability enhanced overall recall for simple strategy users, but not complex strategy users; and that greater levels of numeric ability attenuated the amount of information forgotten over 7-days, but only for those individuals who used the most complex strategies. There is extensive training research that has employed mnemonic procedures, including the number-consonant mnemonic, and has demonstrated improvements in the recall of numeric information as a consequence [17]. Training studies should evaluate baseline numeric ability as a marker of strategy use and, potentially, whether an individual will benefit from learning and employing mnemonic strategies to facilitate number recall.
